# *ATG16L1* and *OPTN* as a novel prognostic gene expression signature in acute myeloid leukemia survival

**DOI:** 10.3389/fonc.2026.1784384

**Published:** 2026-05-05

**Authors:** Alexandra Teixeira, João M. Alves, Nuria Estévez-Gómez, Ângela Fernandes, Nuno Cerveira, Susana Bizarro, Manuel R. Teixeira, Manuel Guerreiro, Isabel Castro, Belém Sampaio-Marques, José Mariz, Sara Abalde-Cela, Lorena Diéguez, David Posada, Paula Ludovico

**Affiliations:** 1 International Iberian Nanotechnology Laboratory (INL), Braga, Portugal; 2 Life and Health Sciences Research Institute (ICVS), Escola de Medicina, Universidade do Minho, Braga, Portugal; 3 ICVS/3B’s - PT Government Associate Laboratory, Braga, Portugal; 4 Centro de Investigación en Nanomateriais e Biomedicina (CINBIO), Universidade de Vigo, Vigo, Spain; 5 Galicia Sur Health Research Institute (IIS Galicia Sur), SERGAS-UVIGO, Vigo, Spain; 6 Department of Genetics, Portuguese Oncology Institute-Porto (IPO-Porto)/Porto Comprehensive Cancer Center, Porto, Portugal; 7 School of Medicine and Biomedical Sciences (ICBAS), University of Porto, Porto, Portugal; 8 Hospital da Arrábida, Vila Nova de Gaia, Portugal; 9 Department of Onco-hematology, Portuguese Oncology Institute-Porto (IPO-Porto), Porto, Portugal; 10 Department of Biochemistry, Genetics, and Immunology, Universidade de Vigo, Vigo, Spain

**Keywords:** acute myeloid leukemia (AML), *ATG16L1*, autophagy, OPTN, overall survival (OS)

## Abstract

Acute myeloid leukemia (AML) is the most frequent type of leukaemia in adults, often with poor outcomes due to the complex genetic landscape and low treatment efficacy. Autophagy, a conserved degradation process, plays an indispensable and context-dependent role in AML. This work explored the prognostic impact of autophagy-related genes compared to known AML-related genes. A two-gene signature (ATG16L1-OPTN) demonstrated that the expression of these autophagy-related genes correlates significantly with overall survival (OS). Cox regression analysis and validation in two larger cohorts (BEATAML2andTCGA LAML) confirmed the association of this signature with worst OS, reinforcing its clinical relevance. These results suggest the ATG16L1-OPTN signature as a novel and robust prognostic marker for predicting OS in AML patients.

## 1 Introduction

Acute myeloid leukemia (AML) is a heterogeneous hematological neoplasia affecting the normal production of blood cells and characterized by the rapid growth and accumulation of immature cells. Despite some encouraging advances in treatment strategies, AML remains a highly fatal disease, with an overall 5-year survival rate around 30% (1–3). Several mutations with diagnostic and prognostic value in AML have been described in myeloid transcription factor and signaling genes, tumor suppressors, DNA methylation, splicing factors, chromatin modifiers, and in the cohesin complex (4). Currently, molecular abnormalities and cytogenetic characteristics identified at diagnosis are still regarded as the most critical prognostic factors. They play a pivotal role in assessing the complete remission rate, disease-free survival rate, and overall survival (OS) rate, as well as in informing evidence-based management strategies for AML. Importantly, autophagy, a cellular degradative process, has also been associated with AML outcome. Although the reduced expression of autophagy-related genes such as *ULK1*, *ATG3*, *ATG4D*, and *ATG5* has been suggested to benefit AML cell survival (5), inhibiting autophagy may also help overcome AML resistance to chemotherapy (6).

Two different gene signatures have been proposed for AML prognosis (7, 8). The LSC17 signature corresponds to a 17-gene signature which includes a set of leukemic stem cell related genes and has been shown to provide accurate prognostic/predictive information to identify AML patients, with the exception of acute promyelocytic leukemia, who do not benefit from standard therapy (7). Overall, the ALFA-0702 trial showed that LSC17 refines genetic risk stratification in AML patients treated intensively. Currently, LSC17 is being evaluated in a Phase 1/2 trial (ISRCTN31682779) for older AML/MDS patients (https://www.isrctn.com/ISRCTN31682779). Another six gene signature for AML prediction is composed by *CASP3, CHAF1B, KLHL24, OPTN, VEGFA*, and *VPS37C*. This signature, which we refer to as “6G”, was recently proposed based on genes found differentially expressed from a total of 546 autophagy-related genes obtained from the Human Autophagy Database and the Molecular Signatures Database,(GO_autophagy, M12441) (8). Although, autophagy has been involved in AML initiation, progression and response to chemotherapy (9, 10), this gene signature does not include major autophagy-related genes such as *ULK1*, *ATG3*, *ATG4D*, and *ATG5*, which reduced expression has been suggested to benefit AML cell survival (5).

Hence, while dysregulation of autophagy has been associated with overall survival and AML patient’s outcome (9, 10), the genes included in both the LSC17 the 6G signatures comprise large sets of genes that are often linked to diverse cellular pathways and the significance of autophagy toward AML prognosis still needs to be better defined.

Thus, we have explored a custom 33-gene panel composed not only of canonical autophagy-related, AML-related and lysosomal biogenesis genes, but also including genes already implicated in AML inititation and prognosis. From this, a two-gene signature (ATG16L1 and OPTN) was identified and validated in two independent cohorts (BEATAML2 and TCGA-LAML) for clinical stratification of AML patients. As frontline therapies for AML (e.g., FLT3 and BCL2 inhibitors) are influenced by autophagy and mitophagy, our signature (*ATG16L1*-*OPTN)*, could serve to predict treatment response.

Taken together, our results shed new light on the value of autophagy-related biomarkers in AML prognosis and offer clinicians a practical and robust tool for early risk stratification and personalized treatment planning.

## 2 Methods

### 2.1 Human bone marrow samples

Bone marrow (BM) samples obtained at diagnosis (2005-2014) from 33 AML patients were provided by the Instituto Português de Oncologia (IPO)-Porto (**Table 1**). BM samples from 17 donors without hematological malignancies, but with coronary artery disease were also collected from the sternum at the beginning of cardiac surgery at the Hospital da Arrábida-Porto and used as controls. All samples were obtained in accordance with the Declaration of Helsinki, with written informed consent provided by all patients. The whole study was approved by the ethics committees of the ICVS and IPO-Porto institutions.

**Table 1:** Clinical features of AML patients and controls in study.

Characteristics	AML (n=33)	Controls (n=17)
Age		
<60 (n; %)	22; 66.7	2; 11.8
≥60 (n; %)	11; 33.3	15; 88.2
Gender		
Male (n; %)	21; 63.6	13; 76.5
Female (n; %)	12; 36.3	4; 23.5
AML Subtype		
M1 (n; %)	3; 10.3	..
M2 (n; %)	2; 6.9	..
M3 (n; %)	9; 31.0	..
M4 (n; %)	5; 17.2	..
M5 (n; %)	2; 6.9	..
M7 (n; %)	1; 3.4	..
AML Dysplasia (n; %)	7; 24.1	..
Insufficient information (n)	4	..
Prognosis Classification		
Favourable (n; %)	11; 37.9	..
Intermediate I (n; %)	8; 27.6	..
Intermediate II (n; %)	2; 6.9	..
Adverse (n; %)	8; 27.6	..
Insufficient information (n)	4	..
Karyotype		
Normal karyotype (n; %)	8; 27.6	..
Abnormal karyotype (n; %)	21; 72.4	..
Insufficient information (n)	4	..
White Blood Cell count		
<50 × 10^3^/µL (n; %)	24; 77.4	..
≥50×10^3^/µL (n; %)	7; 22.6	..
Insufficient information (n)	2	..
Complete Remission (after first induction)		
Yes (n; %)	24; 72.7	..
No (n; %)	9; 27.3	..
Type of Leukemia		
De novo AML (n; %)	29; 87.9	..
Therapy AML (n; %)	4; 12.1	..
Treatment		
Intensive (n; %)	31; 93.9	..
Best Supportive Care (n; %)¤	2; 6.1	..

### 2.2 Total RNA extraction

Mononuclear cells (MNCs) were isolated with a standard density gradient centrifugation using Histopaque^®^ (Sigma-Aldrich^®^). Posteriorly, MNCs were washed once with phosphate-buffered saline (PBS) solution, followed by erythrocyte lysis with a lysis buffer (8.3 g of NH_4_Cl, 1 g of KHCO_3_, 1.8 mL of 5% EDTA and complete with dH_2_O to a final volume of 1000 mL) and centrifuging at 250 ×g for 10 min. Part of these cells were used to isolate mRNA. Total RNA extraction was performed by NZYol™ RNA Isolation Reagent (nZytech^®^). 100 µL of chloroform was added to each tube, mixed by inverting the tube for approximately 15 s and allowed to rest 3 min at 4 °C. Afterwards, the samples were centrifuged at 13.000 rpm, for 15 min, at 4°C. The resulting upper aqueous phase was removed and transferred into a new tube, avoiding the interphase (containing DNA, proteins and lipids). Then, 250 µL of isopropanol were added and the tube was mixed by inversion and allowed to rest at 4°C for 10 min. The samples were centrifuged as previously described and the supernatant was removed. The pellet was washed with ice cold 70% ethanol. Posteriorly, ethanol was removed and the samples were allowed to dry. The RNA pellets were stored at -80˚C in an RNA stabilizing solution (RNAlater^®^ - ICE Frozen Tissue Transition Solution, Life technologies^®^).

### 2.3 Targeted RNA-sequencing

Total RNA was quantified with the Qubit RNA HS assay kit (Thermo Fisher Scientific) and diluted to 100 ng/µL. The quality of mRNA was confirmed by Bioanalyzer (Agilent Technologies). Samples with RIN≥5.6 were selected for sequencing library preparation with an AmpliSeq™ Custom RNA Panel (Illumina, San Diego, CA). For the targeted RNA-sequencing a new panel was designed by the authors of the present work, composed by a selection of 33 different genes (**Table 2**) mostly composed of autophagy genes, and also AML-related genes. The list of genes included in the AML prognosis encompasses the six-genes proposed by Chen et al. (8). The RNA was polyA selected, fragmented, and randomly primed, and cDNA synthesis was performed using the Ampliseq™ cDNA Synthesis for Illumina^®^ kit, followed by adapter ligation and PCR amplification. Paired-end sequencing with a read length of 150 bp was performed on an Illumina MiniSeq system to at least 2.4 Gb per run, yielding up to eight million reads per run. Image analysis, base calling, and quality checks were also performed.

**Table 2:** Next-generation sequencing (NGS) gene panel.

AML related (6 genes)*	Autophagy related (14 genes)		Lysosomal biogenesis (3 genes)	AML prognosis (8 genes)	Housekeeping genes (2 genes)
*FLT3*	*BCL2*	*SQSTM1*	*CD63*	*CASP3*	*TBP*
*DNMT3A*	*ATG5*	*ULK1*	*LAMP1*	*CHAF1B*	*HMBS*
*IDH1*	*ATG10*	*ULK2*	*CLCN7*	*HDAC1*	
*IDH2*	*ATG16L1*	*ATG9A*		*HIST1H3G*	
*ASXL1*	*ATG3*	*ATG7*		*KLHL24*	
*NPM1*	*ATG14*	*OPTN*		*LAMTOR2*	
	*GABARAP*	*Beclin1*		*VEGFA*	
				*VPS3*	

Genes were selected based on their shown role in AML, as depicted.

### 2.4 RNA-seq data processing and differential expression analysis

Sequencing adapters and low-quality reads (Phred score<15 and sequence lengths<36 bp) were trimmed and removed using Trimmomatic (11). The remaining reads were mapped to the human reference genome Hg38 using STAR (12) under default settings. The output SAM files were sorted by read-name using *samtools sort -n* and used as input for HTSEQ (13) to quantify the number of reads per gene. Raw gene counts were imported into R using the *DESeqDataSetFromHTSeqCount* function from the R package DESeq2 (14) and log-transformed. DESeq2 was used for the calculation of normalized counts for each transcript using default parameters. Differentially expressed genes were identified by comparing AML and control samples using a *p-value* cut-off of 0.05.

### 2.5 Statistical analysis

The prognostic value of gene expression on OS was evaluated with a univariate Cox proportional hazards regression model. Using the genes whose expression showed a statistically significant impact on survival in the univariate analysis, we next fitted a multivariate Cox proportional hazards regression model to describe the survival time as a function of the expression levels. Age and gender were used as covariates. Finally, forest plots were generated using the survival R package (15).

### 2.6 Gene-signature risk scoring system

Patient-specific risk scores were calculated by multiplying the expression level of each gene (Gi) in the signature by its corresponding Cox coefficient (RS = Σ Coef(Gi*)Expression(Gi)) (16). Following a similar approach to Chen et al. (8), AML patients were then classified into high- or low-risk groups using the median cut-off of the risk score as the threshold value. Finally, we used two publicly available, RNA-seq AML datasets-BEATAML2(N = 186) and TCGA-LAML(N = 167)-to validate our gene expression signature *(ATG16L1-OPTN)*. Pre-processed RPKM expression values and clinical details for the TCGA-LAML cohort were obtained from: https://tcga-data.nci.nih.gov/docs/publications/laml_2012/laml.rnaseq.179_v1.0_gaf2.0_rpkm_matrix.txt.tcgaID.txt.gz and https://tcga-data.nci.nih.gov/docs/publications/laml_2012/clinical_patient_laml.tsv, respectively.

Normalized expression counts and clinical data from the BeatAML2.0 cohort were retrieved from: https://biodev.github.io/BeatAML2/. Patients from the two datasets were independently stratified into high- or low-risk groups using the same exact strategy used for our in-house cohort.

### 2.7 Comparison against other prognostic gene signatures

The prognostic value of the *ATG16L1-OPTN* expression signature derived from our analysis was compared against two previously proposed gene-based prognostic signatures in AML: LSC17 – which corresponds to a 17-gene signature related to stemness (7), and a six autophagy-related gene signature (8), which we refer to as “6G”. The patient-specific risk scores for each signature were calculated as described in the previous section.

## 3 Results

### 3.1 Differential gene expression between AML patients and controls

**Figure 1:**
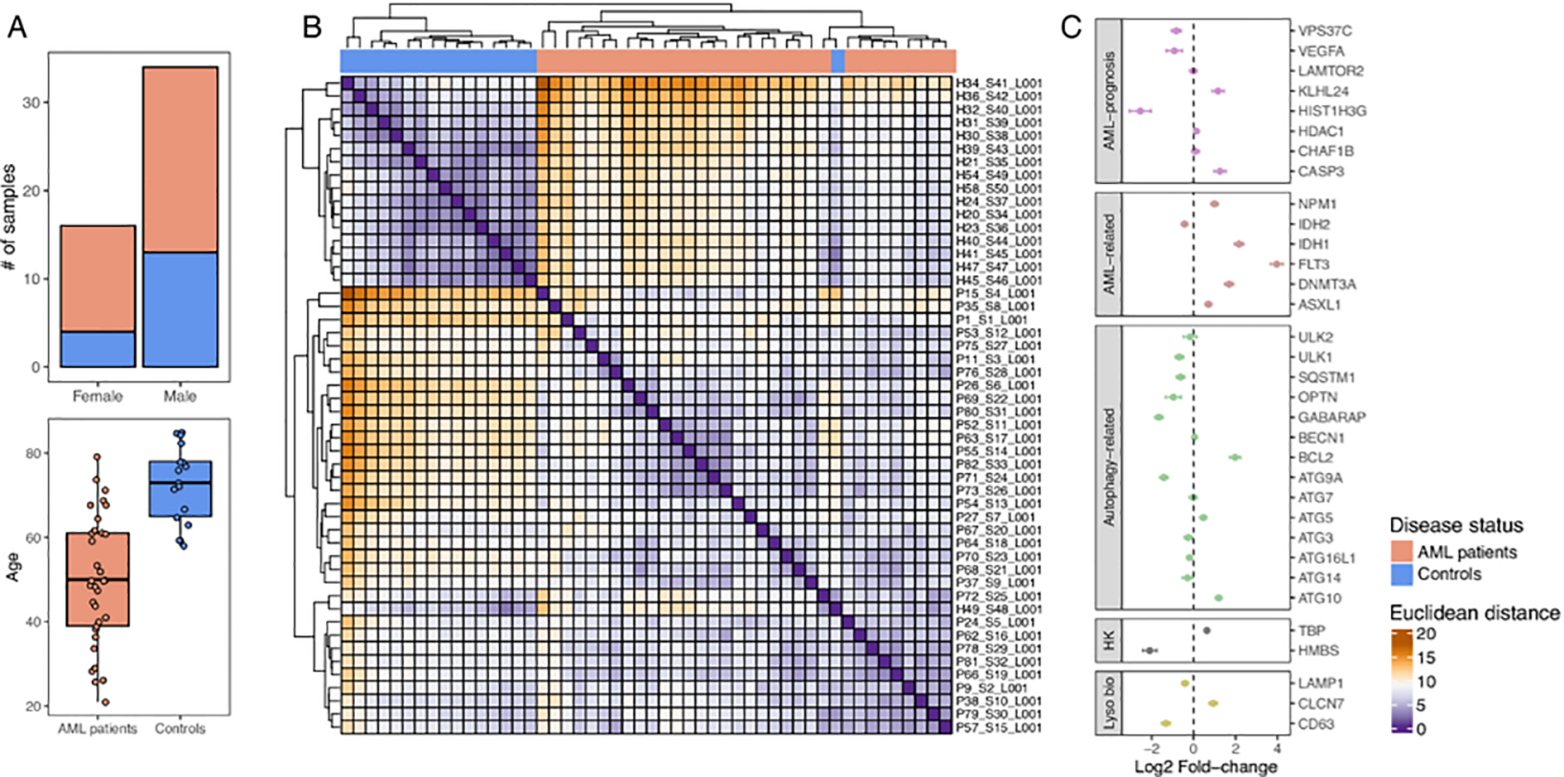
Gene-expression patterns of our in-house cohort. **(A)** Age and gender in our cohort. **(B)** Heatmap describing the sample-to-sample Euclidean distance based on the expression of the 33 genes in our panel across individual samples. The superior axis shows samples classified according to disease status (i.e., AML patients vs. controls). **(C)** Boxplot depicting the differential gene expression (log2 fold-change) between AML patients and control samples.

Targeted RNA-sequencing data were analyzed from bone marrow (BM) samples of 33 AML patients and 17 donors (controls without hematological malignancy). Our in-house cohort was mostly composed of males, with AML patients typically younger than healthy donors (**Figure 1A**). More precisely, AML patient ages ranged from 28 to 79 years, with a mean of 50 years. On the other hand, controls ranged from 59 to 85 years of age, with a mean of 72 years (**Table 1**, Materials and Methods).

Differences in expression between AML patients and controls were found for several genes (**Figure 1B**), independently of the clustering method used (**Supplementary Figure 1**).

We have analyzed 33 genes distributed for different categories as: canonical autophagy-related (*BCL2, SQSTM1, ATG5, ULK1, ATG10, ULK2, ATG16L1, ATG9A, ATG3, ATG7, ATG14, OPTN, GABARAP, Beclin1*); AML-related (*FLT3, DNMT3A, IDH1, IDH2, ASXL1, NPM1);* lysosomal biogenesis *(CD63, LAMP1* and *CLCN7*) with *LAMP1* previous considered a risk factor for AML disease progression (17); previous implicated in AML prognosis (*CASP3, CHAF1B, HDAC1, HIST1H3G, KLHL24, LAMTOR2, VEGFA, and VPS3*); and housekeeping (*TBP* and *HMBS*) (**Table 1**) in both in-house AML patients cohort and control cohort. Notably, *CASP3, CHAF1B, KLHL24, OPTN* and *VEGFA* were also included in the six-genes signature proposed by Chen et al. (8). As expected, the AML-prognostic (*CASP3* and *KLHL24)* (18) and AML-related genes used for AML diagnosis and classification (*FLT3, DNMT3A*, and *NPM1)* were upregulated (18–21), while *HIST1H3G, VEGFA* and *VPS37C* exhibited lower expression in AML patients (8, 22, 23) (**Figure 1C**).

Our results showed that the autophagy-related genes *ATG10*, *ATG5* and *BCL2* were overexpressed, while *SQSTM1* and *OPTN* were under expressed in AML patients (**Figure 1C**), corroborating multiple previous reports (8, 24–27). Moreover, our AML cohort exhibited a down-regulation of *ULK1* (28), and an up-regulation of *ASXL1* (29), but no major differences were found for *ULK2* (8, 28) (**Figure 1C**) when compared with control cohort. Noticeable, our analysis identified *ATG9A*, a crucial element of the autophagy initiation cascade (30) and still largely overlooked in the context of AML, as being downregulated in AML patients (**Figure 1C**).

### 3.2 Genes with prognostic potential in AML patients

To further assess the prognostic value of these autophagy-related genes specifically within the AML cohort, we performed univariate Cox proportional hazards (CPH) analyses for each of the 33 genes in our target panel, using overall survival (OS) as the clinical endpoint. This analysis was restricted to the AML patients only. Out of the full set, five genes showed a significant association with OS (*ASXL1, ATG16L1, ATG3, OPTN*, and *ULK2*). Among these, *ASXL1* and *ATG16L1* were associated with favorable prognosis (Hazard Ratio (HR) <1), while *ATG3*, *OPTN*, and *ULK2* were associated with worse outcomes (HR >1) (**Supplementary Figure 2**). Notably, four of these differentially expressed genes belong to the core autophagy-associated category (*ATG16L1, ATG3, OPTN*, and *ULK2*), further strengthening the notion of autophagy playing a critical role in the determination of OS.

**Figure 2:**
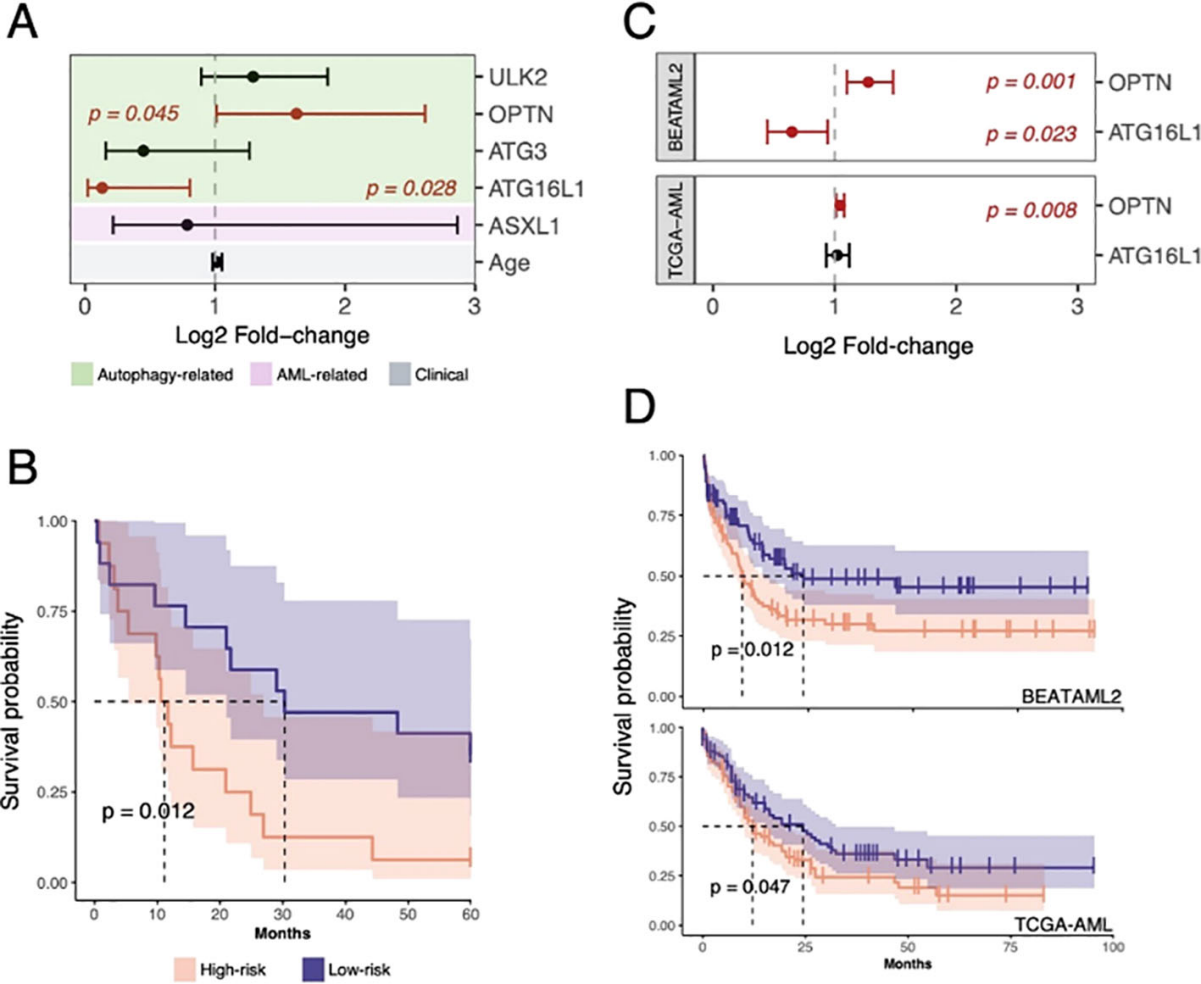
Prognostic value of *ATG16L1* and *OPTN* gene signature in AML. **(A)** Forest plot showing hazard ratios obtained with a multivariate Cox regression for all significant genes in the univariate analysis and age. For the statistically significant genes, the respective bars are colored in red together with their corresponding *p-values*. A dashed vertical line was added at HR = 1. Background colored by variable category. **(B)** Kaplan–Meier’s survival curves describing the difference in survival times between high and low-risk groups according to *OPTN* and *ATG16L1* expression. Dashed lines highlight median survival estimates for each group. **(C)** Forest plot depicting the HR scores obtained by multivariate Cox regression analysis on BEATAML2 and TCGA-AML cohorts. For the statistically significant genes, the respective bars are colored in red together with their corresponding p-values. **(D)** Kaplan–Meier’s survival curves for high- and low-risk subgroups in the BEATAML2 and TCGA-AML datasets. Dashed lines highlight median survival estimates for each group.

In the multivariate CPH analysis, only *ATG16L1* and *OPTN* remained significantly associated with OS in AML patients (*p-value<0.05*; **Figure 2A**). Based on these two genes, we next defined a two-gene prognostic signature (*ATG16L-OPTN*), patient risk scores were calculated as the sum of the expression levels of each gene weighted by their corresponding Cox regression coefficient (see Methods). Using the median score as a cutoff, AML patients were stratified into high-risk (n=16) and low-risk (n=17) groups. The Kaplan-Meier survival analysis revealed that AML patients in the high-risk group had significantly shorter OS compared with those in the low-risk group (HR = 2.7, 95% CI 1.2–6.2, *p-value<0.05*; **Figure 2B**).

Interestingly, a similar trend in OS was observed across the BEATAML2 and TCGA-LAML sets; however, the *OPTN* gene was only significantly associated with OS in the TCGA-LAML set (N = 167) (**Figure 2C**). Nevertheless, a survival analysis showed significant differences in OS between the risk groups defined by our *ATG16L1-OPTN* expression signature in these two datasets (BEATAML2: HR = 1.7, 95% CI 1.1–2.5, *p=0.01*; TCGA-AML: HR = 1.5, 95% CI 1.0–2.2, *p-value=0.04*; **Figure 2D**).

Our *ATG16L1-OPTN* gene expression signature, was also compared against two previously established prognostic models in AML: the “LSC17” (7) and “6G” (8) gene signatures. This comparison was performed in our cohort as well as in BEATAML2 and TCGA-LAML datasets. While the LSC17 signature showed a stronger prognostic power (**Supplementary Figure 3**), the performance of the “6G” and the *ATG16L1-OPTN* signatures was comparable (**Supplementary Figure 4**). These results suggest that the *ATG16L1-OPTN* signature is a robust prognostic marker for OS in AML, offering significant results across multiple independent datasets despite differences in patient demographics, treatment pattern or genetic background. The simplicity of *ATG16L1-OPTN* signature makes it highly practical for clinical implementation, offering a feasible tool for risk stratification in AML.

## 4 Discussion

At present, AML classification and prognosis rely mainly on cytogenetic alterations and specific gene mutations, like *NPM1, FLT3*, and *CEBPA* (18, 31, 32). However, the functional roles of specific genes - including those involved in autophagy - remain unclear in the context of AML, and reliable biomarkers for predicting outcomes are lacking. Suggesting that gene expression profiling, particularly of autophagy-related genes, may provide more relevant prognostic and predictive insights.

The AML-prognostic (*CASP3, CHAF1B, HDAC1, HIST1H3G, KLHL24, LAMTOR2, VEGFA*, and *VPS3*) and AML-related (*FLT3, DNMT3A, IDH1, IDH2, ASXL1, NPM1*) genes exhibited expression patterns in our AML cohort that were consistent with previous studies (8). These genes were analyzed not only to enable comparison with other AML cohorts but also to assess the strength of our gene signature relative to established prognostic markers. Importantly, *ATG9A*, a key component of the autophagy initiation cascade (30), was underexpressed in AML patients, indicating its underexploited role in AML disease.

When evaluating the prognostic value of autophagy-related genes (*ATG16L1, ATG3, OPTN*, and *ULK2*) and the AML-related gene (*ASXL1)*, were found significantly associated with OS in AML patients. Across different AML cohorts evaluated, a multivariate analysis confirmed *ATG16L1* and *OPTN* as significant prognostic factors, highlighting their potential as a robust two-gene signature in AML.

While low *OPTN* expression has already been correlated with better OS (8), *ATG16L1* has never been reported as a protective factor in AML. Compared to existing gene expression signatures for OS prediction in AML (7, 8), the *ATG16L1-OPTN* signature offers potential benefits. The identification of *ATG16L1* and *OPTN* as two genes with potential prognostic relevance may provide a rapid and clinically practical framework for expression-based assessment using established methods such as qPCR, digital PCR, or targeted transcriptomic panels, thereby supporting preliminary therapeutic decision-making before comprehensive molecular profiling is available and avoiding reliance on long multigene signatures that are less easily translated into routine clinical practice. Our findings reinforce the relevance of autophagy in AML survival. Indeed, *OPTN* functions as both an autophagy receptor and an inducer, playing regulatory roles in various stages of the autophagic process, including enhancing autophagosome formation by recruiting the *Atg12-5-16L1* complex (33). Although *OPTN* typically exhibits a protective effect in most autophagy-related diseases, there is an elevated expression of *OPTN* in several tumor types, suggesting a potential tumor suppressor role (34). Autophagy and mitophagy (autophagy of mitochondria) have previously been implicated not only in the OS of AML patients but also in their response to novel therapeutic agents (18). While autophagy can mediate resistance to FLT3 inhibitors (35), increased mitophagy confers resistance to BCL2 inhibitors like venetoclax (36). Since these agents are current frontline therapies (18), and given that autophagy functionality could impact their efficacy, future studies should elucidate the potential of autophagy to predict chemotherapy response in AML patients.

Although further biological investigation and prospective validation are warranted to better clarify the significance of *ATG16L1–OPTN* in AML, our findings suggest that this gene-expression signature may represent a potential marker for risk stratification and deserves further study in relation to therapeutic decision-making.

## Data Availability

The data presented in the study are deposited in the Sequence Read Archive (SRA) database, accession code PRJNA1458280.
