# Supplementary material for: ATG16L1 and OPTN as a novel prognostic gene expression signature in acute myeloid leukemia survival
Source: Front Oncol. 2026 May 5;16:1784384. doi: 10.3389/fonc.2026.1784384 (PMC13184388; doi:10.3389/fonc.2026.1784384)
Supplement: Supplementary file 1 [file DataSheet1.docx]

Supplementary Material

# Supplementary Figures

## Supplementary Figure 1:


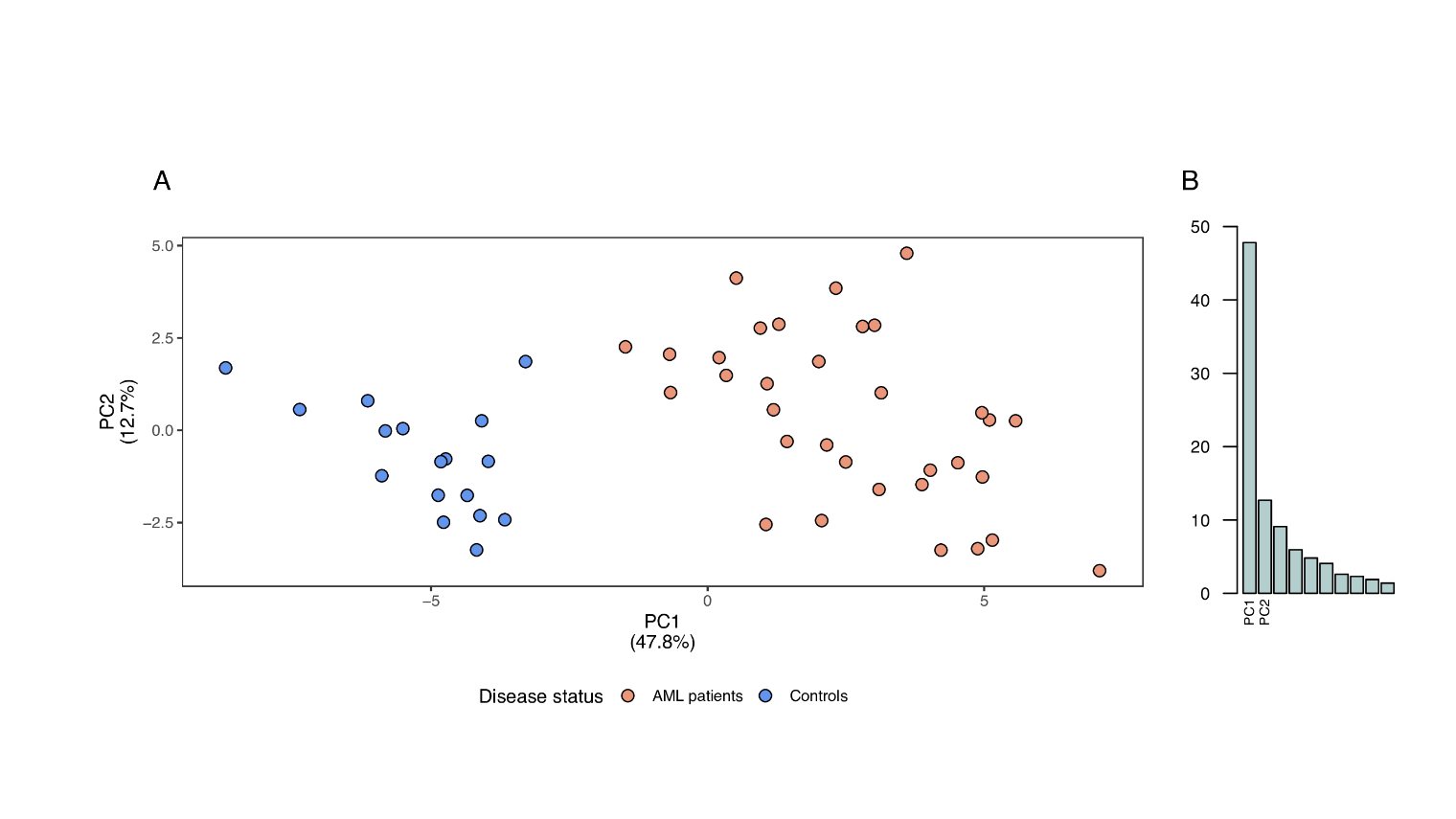


**Supplementary Figure 1. Gene expression differences between AML patients and controls.** (A) Principal component analysis (PCA) of normalized gene counts for each sample. Different colors highlight the disease status of each individual. (B) Bar plot illustrating the fraction of variance explained by each of the first 10 principal components.

## Supplementary Figure 2:


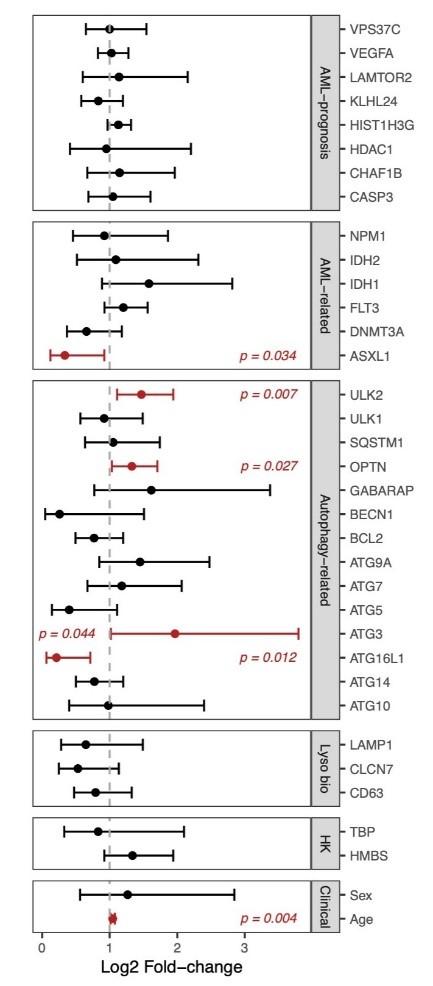


**Supplementary Figure 2.** **Prognostic value of the expression scores of AML- and autophagy-related genes in AML.** Forest plot showing hazard ratios (HR) obtained by univariate Cox regression analysis for each gene in our panel. The age and sex factors were considered in the analysis. Statistically significant genes, along with their respective error bars, are colored in red, and with corresponding *p-value*. A dashed vertical line was added at HR=1 (i.e., no impact on survival).

## Supplementary Figure 3:

***
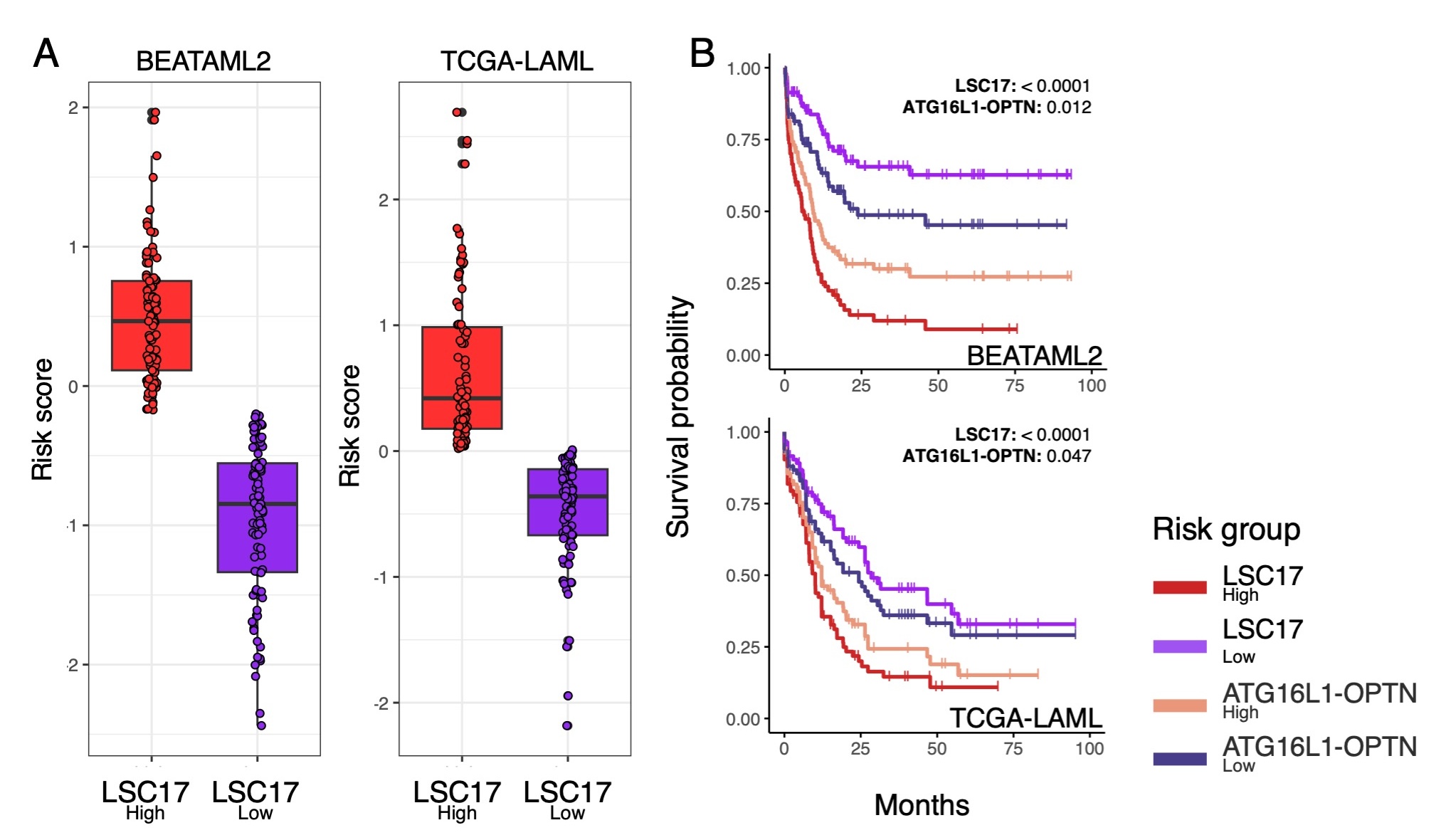
***

**Supplementary Figure 3.** **Comparison between *ATG16L1+OPTN* and LSC17 prognostic signatures.** (A) Patient stratification in BEATAML2 and TCGA-LAML datasets for the LSC17 gene signature. (B) Kaplan-Meier survival curves for the two prognostic signatures in BEATAML2 and TCGA-LAML datasets. Different colors depict the four different groupings. *p-values* for each signature shown above each plot.

## Supplementary Figure 4:

***
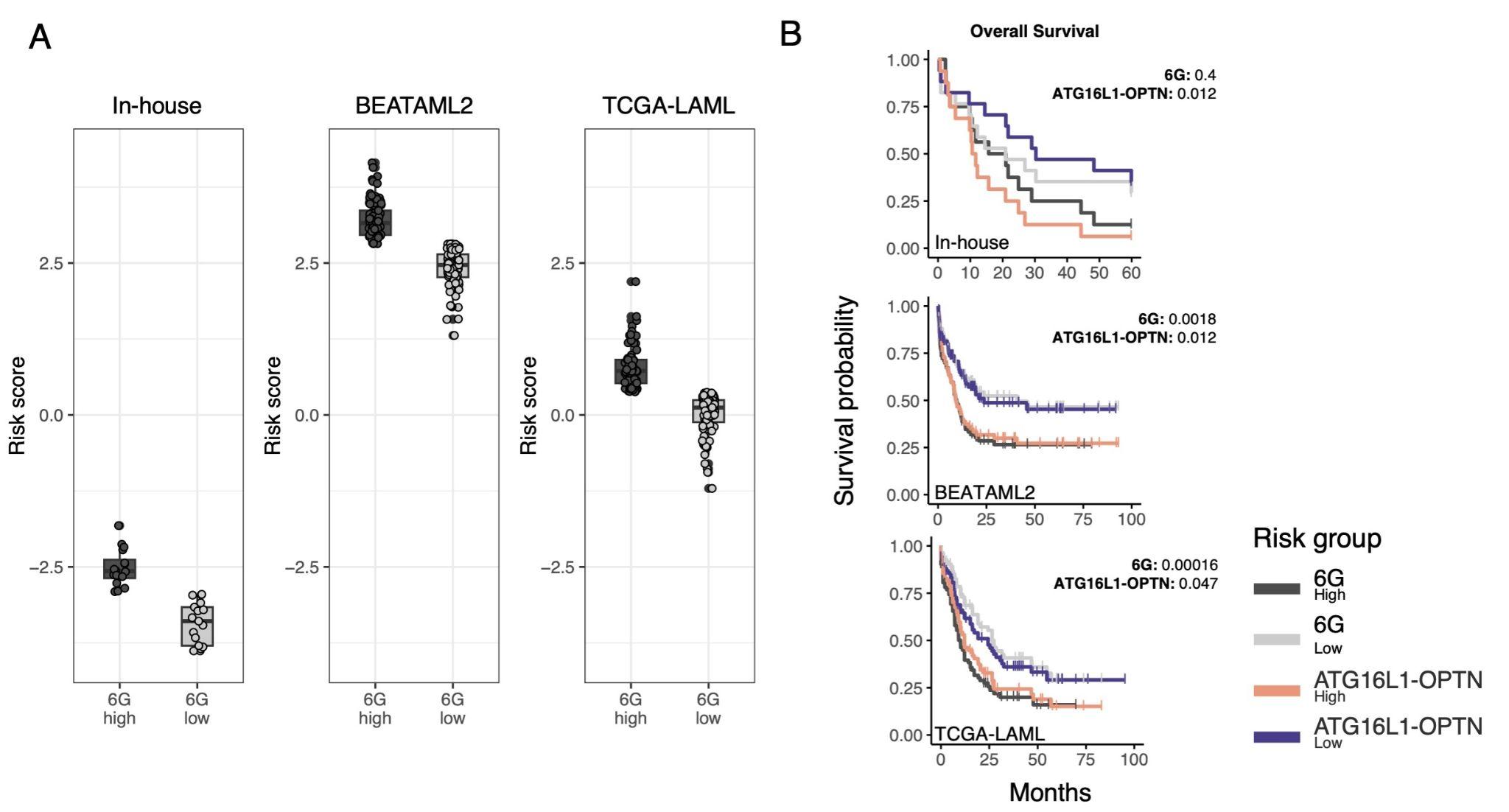
***

**Supplementary Figure 4.** **Comparison between *ATG16L1+OPTN* and 6G prognostic signatures.** (A) Patient stratification in in-house cohort plus BEATAML2 and TCGA-LAML datasets for the 6G gene signature. (B) Kaplan-Meier survival curves for the two prognostic signatures in the three datasets evaluated. Different colors depict the four different groupings. *p-values* for each signature shown above each plot.
